# Molecular Microbial Analysis of *Lactobacillus* Strains Isolated from the Gut of Calves for Potential Probiotic Use

**DOI:** 10.4061/2010/274987

**Published:** 2009-09-01

**Authors:** Lorena P. Soto, Laureano S. Frizzo, Ezequiel Bertozzi, Elizabeth Avataneo, Gabriel J. Sequeira, Marcelo R. Rosmini

**Affiliations:** Departamento de Salud Pública Veterinaria, Facultad de Ciencias Veterinarias, Universidad Nacional del Litoral, Kreder 2805 (S3080HOF) Esperanza, Santa Fe, Argentina

## Abstract

The intestinal microbiota has an influence on the growth and health status of the hosts. This is of particular interest in animals reared using intensive farming practices. Hence, it is necessary to know more about complexity of the beneficial intestinal microbiota. The use of molecular methods has revolutionized microbial identification by improving its quality and effectiveness. The specific aim of the study was to analyze predominant species of *Lactobacillus* in intestinal microbial ecosystem of young calves. Forty-two lactic acid bacteria (LAB) isolated from intestinal tract of young calves were characterized by: Amplified Ribosomal DNA Restriction Analysis (ARDRA), by using *Hae* III, *Msp* I, and *Hinf* I restriction enzymes, and 16S rDNA gene sequencing. ARDRA screening revealed nine unique patterns among 42 isolates, with the same pattern for 29 of the isolates. Gene fragments of 16S rDNA of 19 strains representing different patterns were sequenced to confirm the identification of these species. These results confirmed that ARDRA is a good tool for identification and discrimination of bacterial species isolated from complex ecosystem and between closely related groups. This paper provides information about the LAB species predominant in intestinal tract of young calves that could provide beneficial effects when administered as probiotic.

## 1. Introduction

The natural microbiota of the gastrointestinal tract has an influence on the biochemistry, immunology, physiology, and nonspecific host's resistance against infectious diseases [1]. Therefore, the role of the intestinal microbiota is of vital importance in the nutritional status of the host, and particularly in farm animals that are reared in intensive systems [2]. Because of this it is necessary to determine the complexity of the intestinal flora and recognize the different microorganisms that compose it. This is particularly relevant in the probiotic therapy field where it is necessary to distinguish between probiotics and autochthonous microbiota [3]. 

Lactobacilli are part of the normal human gastrointestinal microbiota and may also be found in other mammalian species [4–7] and birds [8]. It has been reported that some *Lactobacillus* species have probiotic properties and that they are “live micro-organisms which when administered in adequate amounts confer a health benefit on the host” [9].

The first step in the probiotic production is the isolation and identification of the normal components of the gut microbiota, because one of the desirable characteristics of strains used as probiotics is that they should be autochthonous to the ecosystem of which they will be part once ingested [2]. Then, we must assess the probiotic and technological properties of the strains [4] in order to select the best examples that will form the probiotic inoculum. The inocula can be either monostrain or multistrain [10]. The latter is more effective because it can use the complementary and synergistic effects of each microorganism [11].

To analyse and rapidly identify bacteria from microbial communities, classical physiological and biochemical tests are not adequate because the bacterial populations involved often have similar nutritional requirements and grow under similar environmental conditions. Currently, there is a wide variety of molecular strategies, such as PCR with specific primers, DGGE, RAPD, PFGE, FISH, RFLP, and PCR-ARDRA, among others [12], which are available to determine the species diversity of *Lactobacillus* [13].

The comparison of sequences of the 16S rDNA gene is a very reliable method for sorting and identifying bacterial species [14]. Because these genes are highly conserved and are present in large numbers of copies within each bacterial cell, their use as a molecular target has increased in the recent years [15].

The ARDRA technique is a highly discriminatory method, simple and quick to identify Gram positive nonspore bacteria. Many authors have shown that this method is suitable for the discrimination of different species of *Lactobacillus* [8, 16, 17]. In addition, many LAB used as starters or probiotics have been identified with the ARDRA methodology [18].

The aim of this study was to analyse the predominant species of *Lactobacillus* that constitute the intestinal microbial ecosystem of young calves, by means of isolating and identifying strains through the application of the ARDRA technique and 16S rDNA gene sequencing, as a prior step to the design of a probiotic inoculum for cattle.

## 2. Materials and Methods

### 2.1. Bacterial Isolation

Isolates were taken from the mucosa of cecum and jejunum of six young calves reared in intensive conditions. For this, a selective *Lactobacillus* Anaerobic MRS broth with Vancomycin and Bromocresol green (LAMVAB, 7) was used. Forty-one colonies were multiplied in MRS broth for 24 hours at 37°C. For preservation, the cultures were frozen at −80°C with the addition of glycerol 25% v/v.

### 2.2. DNA Isolation

An aliquot of 2 mL of each 24 hours culture was centrifuged at 14000 g (for 5 minutes). The sediment was frozen at −20°C for 24 hours to facilitate the breaking of the cells. The DNA was extracted according to Marmur [19] modified by Kurzak et al. [20] and then resuspended in 50 *μ*L of TE buffer (10 mM Tris-HCl, 1 mM EDTA, pH 8). An aliquot of 5 *μ*L of this template DNA was added directly to the PCR tube. The amount of DNA obtained was quantified by measuring it in an UV spectrum (260 nm) and its integrity was visualised by agarose gel electrophoresis to 0.7% w/v, by staining with ethidium bromide and visualising under UV light.

### 2.3. 16S rDNA Amplification

The 16S rDNA gene was amplified by PCR with a thermal cycler (MJ Research). DNA fragments of approximately 1.5 kpb were amplified using the primers 27F (5′-AGAGTTTGATCCTGGCTCAG-3′) and 1492R (5′-GGYTACCTTGTTACGACTT-3′). Each PCR tube (50 *μ*L) contained a reaction mix of 10 *μ*L 5X PCR buffer for *Taq * polymerase (Promega), 1.5 mM MgCl_2_, 200 *μ*M of each deoxynucleotide triphosphate (Promega), 0.4 *μ*M of each primer and 2 U of *Taq* Polymerase (Promega) and 5 *μ*L of template DNA. The termocycle programme was as follows: 94°C for 5 minutes; 30 cycles of 94°C for 1 minute, 55°C for 1 minute and 72°C for 1 minute; and a final extension step at 72°C for 7 minute. After cycling, the PCR products were visualised by electrophoresis on a 1% w/v agarose gel (40 minute, 75 V), by staining with ethidium bromide (0.5 *μ*g/mL) and visualising under UV light (DyNA Light UV Transilluminator, LabNet, UV light source wavelength 302 nm).

### 2.4. ARDRA

In order to achieve complete digestion, restriction mixes (20 *μ*L of final volume) were carried out for 4 hours at 37°C. Each reaction tube contained 2 *μ*L of 10X incubation buffer, 0.2 *μ*L of bovine serum albumin, 6 U of the respective restriction enzyme, 2.5 *μ*L of bidistilled water and 15 *μ*L of PCR product. Three restriction enzymes were used: *Hae* III, *Msp* I and *Hinf * I (Promega). The resulting digestion products were visualised under UV-light (LabNet Transilluminator, UV light source wavelength 302 nm), after agarose gel electrophoresis 3% w/v (90 minutes, 75 V) by staining with ethidium bromide (0.5 *μ*g/mL). Restriction patterns identical to the sequenced strains led to the identification of the corresponding species [17].

### 2.5. In Silico Study

For this study, Nebcutter software testing protocols (http://tools.neb.com/NEBcutter2/index.php) were used. The theoretical restriction profiles of the 16S rDNA sequence of each species, which had a high percentage of identity in the alignment of the BLAST algorithm, were compared with profiles of the isolates in this study. Besides, theoretical restriction profiles of the 16S rDNA gene sequences were obtained from other species of *Lactobacillus* and *Enterococcus* to determine the power of the ARDRA technique to discriminate from other species.

### 2.6. Sequencing

The PCR products of 19 representative strains of each restriction group were purified with the Wizard PCR SV Gel & PCR Clean-Up System kit (Promega) and sequenced. The sequences were compared with the sequences deposited in the GenBank database using the BLAST algorithm (http://www.ncbi.nlm.nih.gov/BLAST/; 1).

### 2.7. Nucleotide Sequence Accession Numbers

The sequences were deposited in the GenBank database using the web-based data submission tool, BankIt (http://www.ncbi.nlm.nih.gov/BankIt, 1).

## 3. Results

### 3.1. Identification of LAB Isolates by ARDRA

**Figure 1:**
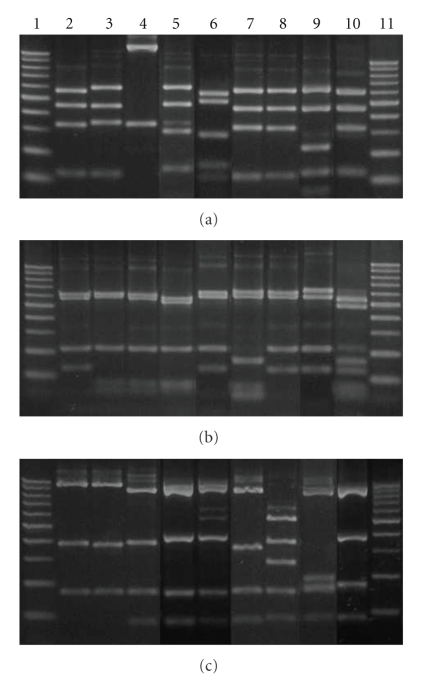
Agarose gel with different groups of restriction. Line 1 and 11, MW ladder (100 bp); line 2, ARDRA group 1 (*L. plantarum*); line 3, ARDRA group 2 (*P. acidilactici*); line 4, ARDRA group 3 (*W. paramesenteroides*); line 5, ARDRA group 4 (*L. salivarius*); line 6, ARDRA group 5 (*L. ruminis*); line 7, ARDRA group 6 (*L. curvatus*); line 8, ARDRA group 7 (*L. farciminis*); line 9, ARDRA group 8 (*L. mucosae*); line 10, ARDRA group 9 (*E. hirae*). Restriction fragments obtained with each enzyme: (a) *Hae* III, (b) *Msp * I, (c) *Hinf * I.

Lactic acid bacteria isolated from calves' intestinal tract samples yielded nine unique ARDRA patterns among the 42 isolates tested (Figure 1). One ARDRA pattern clearly dominated the samples, accounting for 29 of the 42 colonies tested. The other ARDRA patterns from the isolated bacteria were present at a low frequency (Table 2). Most of the ARDRA patterns derived from lactobacilli. Although the isolation medium was specific for *Lactobacillus* spp., two of the patterns found belonged to *Enterococcus* spp.

The restriction of the amplified fragment of the 16S rDNA gene with *Hae* III generated six different profiles. *Lactobacillus plantarum, Weissella paramesenteroides, L. salivarius, L. ruminis* and *L. mucosae* presented specific profiles for each of these species. Instead, *Pediococcus acidilactici, L. farciminis, L. curvatus* and *E. hirae* showed restriction fragments different from the species listed above but not distinguishable among them (Figure 1(a)).

The enzyme *Msp* I also showed six different restriction profiles. Species that showed characteristic profiles were: *L. salivarius, L. curvatus, L. mucosae* and *E. hirae*. It was not possible to differentiate between *P. acidilactici* and *W. paramesenteroides* and between *L. plantarum, L. ruminis * and* L. farciminis* (Figure 1(b)).

*Hinf* I produced seven restriction profiles, five of which were typical of *L. ruminis, L. curvatus, L. farciminis, L. mucosae* and *E. hirae*. The restriction profiles produced by the species *L. plantarum* and *P. acidilactici*, and by *W. paramesenteroides* and *L. salivarius* were not able to distinguish between them (Figure 1(c)).

The restriction profile of each isolate and its association with the concerned species are detailed in Table 1.

### 3.2. In Silico Study

The size of the fragments obtained by the theoretical restriction of the sequences obtained from GenBank that had a higher percentage of identity with the isolations coincided with the restriction fragments obtained in the in vitro study.

On the other hand, some nonisolated species that belonged to the same genus or phylogenetic group as that of the isolates were distinguishable with the *in silico* study, in most cases by restriction with the *Hinf* I enzyme (Table 3).

### 3.3. Identification by Sequencing of the 16S rRNA Gene

Nineteen representative clones of the ARDRA profiles observed were selected for sequencing.

The sequences of the gene fragments obtained from the 16S rDNA were aligned with those from GenBank using the BLAST algorithm. Table 1 shows the percentage of identity of the isolated strains in relation to those found in the database and the access number to GenBank for each of the sequences obtained.

We found that the isolates DSPV 322T, 324T, 325T, 327T, 329T, 333T, 340T, 344T, and 355T represented the ARDRA patterns that were observed most frequently (29 times) among the 42 isolates tested, and that their 16S sequence was most closely related to *Lactobacillus salivarius.*

This species was found in all the calves studied. The other species were represented by one, two or three isolates and were found in one or two calves depending on the case. On the other hand, with the exception of *P. acidilactici,* which was found in the jejunum and in the cecum, the species isolated in the large intestine were different from those isolated in the small intestine (Table 2).

## 4. Discussion

The identification of microbial species through the use of phenotypic methods can sometimes be uncertain, complicated and time-consuming. The use of molecular methods has revolutionised their identification, by improving the quality and effectiveness of this identification. Some of these methodologies use either the rDNA spacer region or its target. These techniques are useful for both the identification and reliable detection of different bacterial species as well as the monitoring of the species [21]. In this way, members of a probiotic multistrain inoculum can be identified and distinguished from strains that share the same environment such as starters in foods (yogurt, cheese, etc.).

The use of species-specific primers or probes is not applicable in environments where there are several *Lactobacillus* species because prior knowledge of them is required. In these cases, more general molecular tools should be applied [21]. The techniques used to identify *Lactobacillus* species in different environments are the comparison of total or partial sequences of 16S rDNA, ARDRA patterns of 16S rDNA or the intergenic region of the 16S-23S rDNA [5, 8, 22].

While the use of 16S DNA sequencing methods gives a high resolution of the diversity of microbial species in an environment, it is very time-consuming and too costly to be used for routine screening of samples. Methods for the initial analysis of faecal samples should be rapid and able to give a broad view of the microbial ecology. ARDRA has been used to compare bacterial isolates within a wide range of microbial communities. The advantages of ARDRA are that it is rapid, reproducible, relates to microbial diversity, and will be invaluable in analysing a greater number of samples together with experimental objectives such as dietary interventions [6].

In the present work, ARDRA allowed us to differentiate *Enterococcus hirae* from the rest of the *Lactobacillus* spp. isolates. This differentiation was observed by restricting with any of the three enzymes used.

The *Lactobacillus* isolated belonged to two groups: the *L. casei-Pediococcus* group and the *Leuconostoc* group. The latter includes the species *Weissella* and *Lactobacillus paramesenteroides*, which can be differentiated from the *L. casei-Pediococcus* group by the typical profile obtained with the restriction enzymes *Hae III* and *Hinf I*. This methodology also allowed the distinction between phylogenetically related species belonging to the *L. casei-Pediococcus* group. These species were *L. ruminis*, *L. salivarius*, *L. curvatus*, *P. acidilactici*, *L. farciminis*, *L. plantarum,* and *L. mucosae*, which have a 16S rDNA homology of 90.3 to 99% [23].

The similarity between the profiles obtained by the *in silico* study of the sequences of the GenBank and the isolates revealed that the strains of the same species had similar profiles. This result proved to be another tool for the identification of the species. The possibility to obtain these profiles, distinguishable between the isolates, together with the differentiation of these isolates from other related species (Table 3), shows that this technique allows the distinction of species with high homology. Such is the case of *E. faecium* and *E. faecalis,* which were also found in the intestines of calves [7] and can be distinguished from *E. hirae* by the restriction enzyme *Hinf I*. There are species within the same phylogenetic groups, such as *L. fermentum* and *L. reuteri*, which have higher homology than others and are most closely related to *L. mucosae* [24]. Despite these similarities, in the present work the *in silico* study showed that the latter could be distinguished from the first two by the ARDRA methodology (Table 3). These results show that the ARDRA technique is a tool that highly discriminates between LAB species and seems to group the isolates by species and then sequence some exponents of each group. This may save both time and money when it is necessary to analyse large numbers of isolates. 

*L. salivarius* was the predominant species in the gastrointestinal tract of calves. It was found in the cecum of all individuals (Table 2) and in some animals it was the only species isolated. This species was also detected by Schneider et al. [7] in calves reared in the same geographical area. *L. salivarius* is an inhabitant of the gastrointestinal tract of other species such as chickens [8], pigs [25], and humans [4, 5]. Many strains that correspond to this species have been studied to evaluate their probiotic properties. Some strains isolated from infants have shown antimicrobial capacity against pathogens [26], and, in particular, *L. salivarius* CTC2197 was able to prevent the colonization of *Salmonella enteritidis* in chickens [27].

The probiotic properties of microorganisms are characteristic of each strain. Therefore, belonging to a species is not sufficient to guarantee the possession of such properties. For a strain to be used as a probiotic, it should be considered GRAS, that is, possessing probiotic effects and technological capabilities suitable for its propagation and preservation over time. Therefore, in order to select the best specimens, in future works we aim at evaluating the probiotic properties of each isolate obtained in this study (in vitro: aggregation, coaggregation with pathogens, production of inhibitory substances, bile and pH resistance; in vivo: effect on calves performance, challenge with pathogen). The knowledge of such properties will allow the development of an inoculum for young calves to improve their performance in intensive farming systems.

## Figures and Tables

**Table 1:** List of bacterial isolated in this study and their closest affiliation according to the 16S rDNA sequencing (1500 pb) or by belonging to the same ARDRA group.

Lactobacilli isolates	ARDRA group	Calf	Specie	Identity value	Accession number
DSPV 320T	9	1	*Enterococcus hirae*	98%	FJ751777
DSPV 321T	8	1	*Lactobacillus mucosae*	99%	FJ751778
DSPV 322T	4	1	*Lactobacillus salivarius*	99%	FJ751779
DSPV 323T	4	1	*Lactobacillus salivarius*		
DSPV 324T	4	1	*Lactobacillus salivarius*	99%	FJ751780
DSPV 325T	4	1	*Lactobacillus salivarius*	95%	FJ751781
DSPV 326T	4	2	*Lactobacillus salivarius*		
DSPV 327T	4	2	*Lactobacillus salivarius*	99%	FJ751782
DSPV 328T	4	2	*Lactobacillus salivarius*		
DSPV 329T	4	2	*Lactobacillus salivarius*	99%	FJ751783
DSPV 330T	4	2	*Lactobacillus salivarius*		
DSPV 331T	4	2	*Lactobacillus salivarius*		
DSPV 332T	4	2	*Lactobacillus salivarius*		
DSPV 333T	4	3	*Lactobacillus salivarius*	99%	FJ751784
DSPV 334T	4	3	*Lactobacillus salivarius*		
DSPV 335T	4	3	*Lactobacillus salivarius*		
DSPV 336T	4	3	*Lactobacillus salivarius*		
DSPV 337T	4	3	*Lactobacillus salivarius*		
DSPV 338T	4	3	*Lactobacillus salivarius*		
DSPV 339T	4	3	*Lactobacillus salivarius*		
DSPV 340T	4	4	*Lactobacillus salivarius*	99%	FJ751785
DSPV 341T	4	4	*Lactobacillus salivarius*		
DSPV 342T	4	4	*Lactobacillus salivarius*		
DSPV 343T	5	4	*Lactobacillus ruminis*	99%	FJ751786
DSPV 344T	4	5	*Lactobacillus salivarius*	99%	FJ751787
DSPV 345T	4	5	*Lactobacillus salivarius*		
DSPV 346T	9	5	*Enterococcus hirae*	98%	FJ751788
DSPV 347T	1	5	*Lactobacillus plantarum*		
DSPV 348T	2	5	*Pediococcus acidilactici*	99%	FJ751789
DSPV 349T	3	5	*Weissella paramesenteroides*	90%	FJ751790
DSPV 350T	2	5	*Pediococcus acidilactici*		
DSPV 351T	3	5	*Weissella paramesenteroides*		
DSPV 352T	6	5	*Lactobacillus curvatus*	99%	FJ751791
DSPV 353T	7	5	*Lactobacillus farciminis*	94%	FJ751792
DSPV 354T	1	5	*Lactobacillus plantarum*	99%	FJ751793
DSPV 355T	4	6	*Lactobacillus salivarius*	99%	FJ751794
DSPV 356T	4	6	*Lactobacillus salivarius*		
DSPV 357T	4	6	*Lactobacillus salivarius*		
DSPV 358T	2	6	*Pediococcus acidilactici*	99%	FJ751795
DSPV 359T	4	6	*Lactobacillus salivarius*		
DSPV 360T	4	6	*Lactobacillus salivarius*		
DSPV 361T	4	6	*Lactobacillus salivarius*		

**Table 2:** Number of isolates for each ARDRA group; frequency of occurrence of each species and portion of the intestine in which the isolates were obtained.

ARDRA group^(a)^	Related species	Isolates^(b)^	Frequency^(c)^	Portion of intestine
4	*L. salivarius*	28/42	6/6	Cecum
2	*P. acidilactici*	3/42	2/6	Cecum/jejunum
6	*L. curvatus*	1/42	1/6	Cecum
1	*L. plantarum*	2/42	1/6	Jejunum
7	*L. farciminis*	1/42	1/6	Jejunum
9	*E. hirae*	2/42	2/6	Cecum
3	*W. paramesenteroides*	2/42	1/6	Jejunum
5	*L. ruminis*	1/42	1/6	Cecum
8	*L. mucosae*	1/42	1/6	Cecum

^(a)^The numbers correspond to the ARDRA groups of the agarose gel electrophoresis (Figure 1).

^(b)^Isolates: number of isolates for each group/total isolates.

^(c)^Frequency: number of calves in which each species was isolated/total number of calves studied.

**Table 3:** *In silico* study.

Phylogenetic group	Isolated species	Related species^(a)^	Enzymes^(b)^
*Enterococcus * group	*E. hirae*	*E. faecium*	*Hinf* I
		*E. faecalis*	*Hinf * I
		*E. lactis*	*Hinf * I
		*E. sanguinicola*	*Hinf * I
		*E. thailandicus*	*Hinf* I

*Leuconostoc * group	*W. paramesenteroides*	*Leuconostoc paramesenteroides*	*Hae * III
		*W. confusa*	*Hinf * I
		*W. minor*	*Hinf * I
		*W. viridenses*	*Hinf * I

*L. casei-Pediococcus * group	*L. mucosae*	*L. fermentum*	*Hinf * I and * Msp * I
		*L. reuteri*	*Hinf * I and * Msp * I
	*L. salivarius*	*L. mali*	*Msp * I
	*P. acidilactici*	*P. pentosaceus*	*Hinf * I
	*L. curvatus*	*L. casei*	*Hinf * I
		*L. sakei*	*Hinf * I
		*P. parvolus*	*Hinf * I

^(a)^Species related with the isolates using the BLAST algorithm, and that differ in the restriction of the 16S rDNA gene profiles.

^(b)^Enzymes for differentiating species isolated from related species.
